# Vector-borne disease and climate change adaptation in African dryland social-ecological systems

**DOI:** 10.1186/s40249-019-0539-3

**Published:** 2019-05-27

**Authors:** Bruce A. Wilcox, Pierre Echaubard, Michel de Garine-Wichatitsky, Bernadette Ramirez

**Affiliations:** 1 ASEAN Institute for Health Development, Mahidol University, 999 Salaya Phuttamonthon, Nakon Pathom, 73170 Thailand; 2 ASTRE, Université de Montpellier, CIRA, INRA, F-34398 Montpellier, France; 3 Faculty of Veterinary Sciences, Kasetsart University, Bangkok, Thailand; 4 Special Programme for Research and Training in Tropical Diseases, World Health Organization, Geneva, Switzerland

**Keywords:** Vector-borne diseases, Integrated vector management, Complexity, Social-ecological system, Biodiversity, Resilience, Climate change adaptation, Traditional knowledge, Adaptive vector borne disease management

## Abstract

**Background:**

Drylands, which are among the biosphere’s most naturally limiting and environmentally variable ecosystems, constitute three-quarters of the African continent. As a result, environmental sustainability and human development along with vector-borne disease (VBD) control historically have been especially challenging in Africa, particularly in the sub-Saharan and Sahelian drylands. Here, the VBD burden, food insecurity, environmental degradation, and social vulnerability are particularly severe. Changing climate can exacerbate the legion of environmental health threats in Africa, the social dimensions of which are now part of the international development agenda. Accordingly, the need to better understand the dynamics and complex coupling of populations and environments as exemplified by drylands is increasingly recognized as critical to the design of more sustainable interventions.

**Main body:**

This scoping review examines the challenge of vector-borne disease control in drylands with a focus on Africa, and the dramatic, ongoing environmental and social changes taking place. Dryland societies persisted and even flourished in the past despite changing climates, extreme and unpredictable weather, and marginal conditions for agriculture. Yet intrusive forces largely out of the control of traditional dryland societies, along with the negative impacts of globalization, have contributed to the erosion of dryland’s cultural and natural resources. This has led to the loss of resilience underlying the adaptive capacity formerly widely exhibited among dryland societies. A growing body of evidence from studies of environmental and natural resource management demonstrates how, in light of dryland system’s inherent complexity, these factors and top-down interventions can impede sustainable development and vector-borne disease control. Strengthening adaptive capacity through community-based, participatory methods that build on local knowledge and are tailored to local ecological conditions, hold the best promise of reversing current trends.

**Conclusions:**

A significant opportunity exists to simultaneously address the increasing threat of vector-borne diseases and climate change through methods aimed at strengthening adaptive capacity. The integrative framework and methods based on social-ecological systems and resilience theory offers a novel set of tools that allow multiple threats and sources of vulnerability to be addressed in combination. Integration of recent advances in vector borne disease ecology and wider deployment of these tools could help reverse the negative social and environmental trends currently seen in African drylands.

**Electronic supplementary material:**

The online version of this article (10.1186/s40249-019-0539-3) contains supplementary material, which is available to authorized users.

## Multilingual abstracts

Please see Additional file 1 for translations of the abstract into the five official working languages of the United Nations.

## Background

**Fig. 1:**
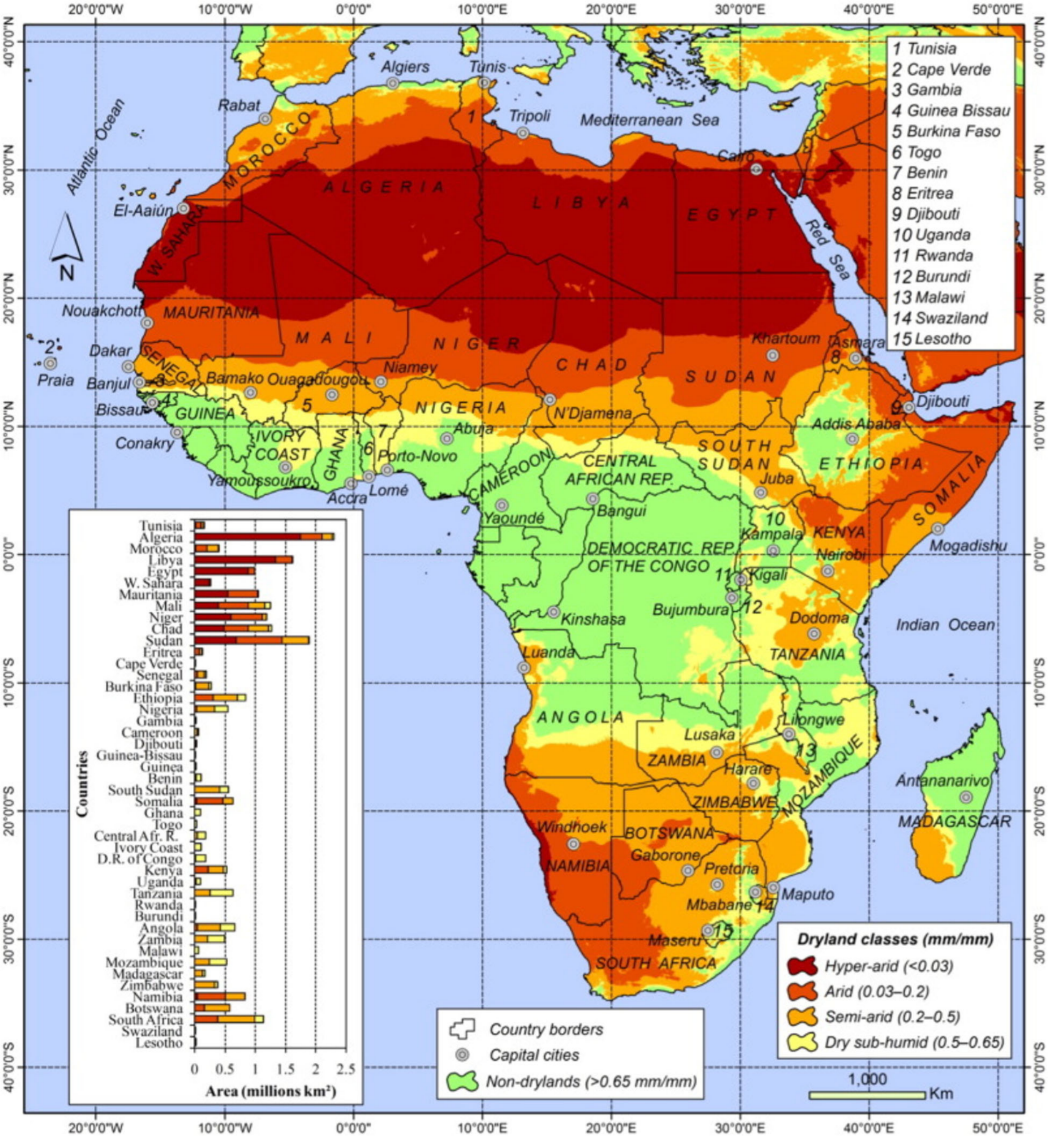
Map of Africa delineating drylands. This shows the geographic distributions of each of the four dryland types: hyper-arid, arid, semi-arid, and dry sub-humid. Each of these zones exhibits the characteristics inherent to drylands described in the text, including naturally greater climate variability than other biomes, However, Hyper-arid and Arid zones both naturally exhibit more and increasingly extreme climate and environmental conditions, including climate variability, which are expected to increase in the coming decades [2]

Africa is recognized as particularly challenging in terms of human development progress among the world’s developing regions [1]. Of the variety of political, economic, and environmental reasons, the continent’s disproportionate extent of drylands [2] and vector-borne diseases are major contributing factors [3]. Drylands, which include hyper-arid to dry sub-humid climate zones (Fig. 1) are naturally characterized by dust storms, temperature extremes, variable rainfall and drought, low agricultural productivity, and zoonotic and vector-borne disease emergence [2]. These natural hazards, already exaggerated in drylands, are exacerbated anthropogenically through deforestation and land degradation [4], dams and irrigation projects [5], pesticide and anti-microbial use, vector and pathogen resistance [6], and political conflict [7]. Adding to this, global climate change is predicted to contribute to increasing climate extremes and drought severity in African drylands [8]. The increased climate variability will further challenge conventional vector borne disease control efforts and require adaptive approaches that include, among other tools, novel meteorological forecasting platforms [9].

Application of the new understandings of environmental change and human adaptation recently generated by interdisciplinary studies examining social and ecological dimensions and their linkages in dryland systems will also be required. Among these, those employing integrative, ecosystem-oriented approaches and sustainability science offer a promising alternative to conventional drylands development approaches of the past, and renewed hope for reversing the above trends [10, 11]. Focusing on the integration of dryland peoples’ distinctive livelihoods and ecological circumstances are key elements of these integrative approaches, particularly in recognition of how dryland peoples’ traditional livelihoods include adaptation to extreme climate variability [12, 13]. For example, mobility and migration as a means of diluting risk historically has been a common coping strategy among dryland pastoralists such as the Turkana [14]. Dryland farmers, analogously fine-tuned cropping systems to the varying environment, including drawing on dryland’s unique and surprisingly rich *in situ* repositories of traditional crop genetic resources. Thus, dryland peoples’ traditional crop production systems historically have proved highly effective in securing well-being despite harsh conditions [15, 16].

Unfortunately, increasing sedentism, including that due to forced settlement by governments among the Maasai and other pastoralist groups has undermined these strategies, and often with negative nutritional and health consequences [17–19]. However, local and traditional knowledge relevant to coping strategies remains and can be used to help restore adaptive capacity. This has been extensively documented as applicable to rural ecosystems, including drylands (i.e., [20–22]). More recently, the applicability of participatory processes, and traditional knowledge inclusion, specifically in relation to climate change adaptation employing the social-ecological systems frame, has been demonstrated for drylands [12, 16, 23–25].

Social-ecological systems coupling in drylands extends to the complex interplay between environments, vectors of zoonotic parasites (e.g., ticks, fleas, black flies, mosquitoes and sand flies), their relationships with humans, and transmission of bacteria, viruses, protozoa or helminths [26]. Thus social-ecological systems framing is recognized as applicable to the problem of infectious disease emergence in general [27–29], zoonotic and vector-borne diseases in particular [30, 31], and integrated vector management [27, 32] as well as to climate change adaptation [33]. This framing emphasizes local community participation, and often involves an ecosystem-based approach centered on ‘adaptive management’ [34–36].

Vector-borne disease control and climate change adaptation, which clearly co-depend upon sustainability science and its applications [37], span multiple disciplines including but not limited to the biomedical, public health, and environmental sciences. As such, interdisciplinary and transdisciplinary approaches employing an integrative framework that can accommodate a social-ecological systems perspective and methods of analysis is required. The area referred to as social-ecological systems theory, with its unique complex systems-based conception of resilience (SESR), is particularly relevant to addressing problems such as pest management (and by extension vector control). SESR represents a large body of research and practical experience applied to environmental and natural resources management problems including pest control. The fundamentals are described in several major works [20, 21, 38] on the basis of which an expansive body of literature has developed outside of the health sciences.

This review examines this body of research and practice as it applies to drylands and how it may provide the basis for an integrative framework for strategies that combine vector-borne diseases and climate adaptation. We conclude with suggestions for going forward with research and methodological development to further operationalize application of the SESR framework. This includes adapting SESR practice to the increasing threats posed by the interplay of vector borne disease and changing climate.

## Main text

### Vulnerability of dryland populations

Dryland rural populations of the developing regions are among the most ecologically, socially and politically marginalized [39]. Their health and economic indices include higher infant mortality and income levels typically among the world lowest [40, 41]. Up to 20% of drylands are ‘desertified’ with their populations historically subject to extreme drought while more frequent droughts are expected due to climate change [9]. Rapid population increase, degradation of the land and its productive capacity, at-risk livelihoods, and migration, including of refugees fleeing environmental conditions or violent conflict, converge in some dryland areas such as the Sahel [11]. Even in the absence of these conditions, dryland peoples’ livelihoods have been among the most negatively impacted by unsustainable development schemes, in particular those associated with agricultural intensification [23].

The expansion into dryland rural areas of ‘modernization’ including changes in land management, appropriation of indigenous societies’ land by governments, development schemes involving ecologically inappropriate and culturally insensitive technologies, such as ill-designed irrigation projects, have been widely documented as contributing to drylands environmental degradation [23, 42]. Less widely studied have been the negative health consequences apparent, for example, in pastoralist populations forced to abandon their traditional practices [19]. These negative health consequences include, for example, higher levels of malnutrition and higher rates of respiratory and diarrheal morbidity in settled versus nomadic communities [19].

In general, dryland populations live under conditions of increasing insecurity due to land degradation and desertification, which tends to worsen as the productive land per capita declines with population growth. The potential for unpredictably changing patterns of vector-borne diseases associated with climate change represents a further challenge for rural populations already facing a range of social and environmental circumstances in constant flux. Ironically, the major concerns about climate change, i.e., weather extremes and climate variability, are nothing new to dryland people who can be said to be masters of adaptation to unpredictable and extreme meteorological conditions [43].

### High impact vector borne diseases in African drylands

Dryland people have co-existed and co-evolved for centuries or more with a range of zoonotic and vector borne diseases some of which, like trypanosomaisis and rinderpest, have been significantly controlled or eliminated. However, many—of which malaria, rift valley fever, typhus and schistosomiasis are most prominent—persist today in spite of decades of intervention programs. These diseases can have significant impacts on livelihoods. Schistosomiasis for instance has profound negative effects on child development, outcomes of pregnancy, and agricultural productivity. Schistosomiasis is thus presented as a key reason why the “bottom 500 million” inhabitants of sub-Saharan Africa continue to live in poverty [44].

The World Organisation for Animal Health has listed a number of High Impact Diseases that must be reported because they can have a significant negative effect on the lives of humans and animals (http://www.oie.int/en/animal-health-in-the-world/oie-listed-diseases-2018/). In arid and semi-arid environments, vector-borne diseases that have a significant impact on livestock include African swine fever, lumpy skin disease, Rift Valley fever and trypanosomiasis, the last two mentioned also having direct pathological effects on humans. Ticks and tick-borne diseases have major impacts on public health and animal health all over the world [45]. They arguably represent the most serious health threat to livestock farmer’s livelihoods in drylands. Direct costs associated with tick-borne diseases include mortality of livestock, due to highly fatal diseases like heartwater, East Coast fever and Corridor disease, and reduced productivity due to erosive diseases such as bovine anaplasmosis. In addition, indirect costs for tick control programmes represent a significant burden for farmers, as the use of synthetic acaricides with harmful residual effects on meat and milk for human and animal consumption is still the primary method of control [46].

### Vector-borne diseases and climate variability challenges unique to drylands

Existing evidence suggests that VBD burdens will increase for people whom are already vulnerable to climate extremes, such as those in the African continent. This is notably pronounced in the dryland areas in the sub-Saharan and Sahelian region [47]. In this region, poor agro-pastoral communities may suffer socio-economically disproportionally more from the effects of changing climate [48, 49], and thus may become more vulnerable to VBD threats.

Drylands’ distinctive biophysical, socio-political and economic circumstances along with their unique vector, pathogen reservoir, and human ecologies produce transmission dynamics and thus a VBD burden specific to these bioclimatic zones [47]. This is a consequence of two sets of characteristics unique to drylands, as distinct from wetter tropical biome types (i.e. tropical forest, woodland and grassland). First, dryland’s high mean and extreme temperatures can result in higher transmission potential of arthropod vectors. This is a consequence of the reduced vector generation time and pathogen incubation period, increased vector population growth rates, and a longer transmission period due to warmer ambient temperatures [50]. Second, dryland’s high seasonal and spatial variation in rainfall results in a more aggregated distribution of primary production. This, in turn, can produce higher VBD transmission rates through increased vector-host interactions, at waterholes and preferred rangeland foraging patches, for example. This has been shown by GPS tracking studies of patterns of spatial contact between tick larvae, livestock, and wildlife hosts [51, 52].

Climate change is expected to further increase the frequency and intensity of extreme weather events, such as floods and droughts, which deserves particular attention in the context of vector-borne diseases for the above reasons [9]. However, diseases will be differentially affected by climate parameters [53]. It is anticipated for instance that mosquito-borne disease risk could increase as a result of the effect of increasingly localized heavy rainfall on vector breeding opportunities as demonstrated for mosquito vectors of Rift valley fever in Southern and Eastern Africa [54] or Malaria [55]. On the other hand, repeated drought conditions encourage the storage of drinking water by local populations for human and livestock consumption. This will in turn increase the number of breeding sites for mosquitoes, such as *Aedes aegypti,* the primary vectors of dengue in endemic areas [56]. Alternatively, increased rainfall variability may have an inhibiting effect on other vectors thus VBDs, for example, leishmaniasis [57].

Drylands are also characterised by socio-economic features which affect transmission dynamics and the burden of VBDs, including livelihood, demography, social organization, and health systems [47]. Semi-arid regions are particularly sensitive to climate variability because the main livelihoods of their people, pastoralism and/or rain-fed agriculture, rely heavily on ecosystem functions associated with primary production such as soil erosion, nutrient cycling, carbon sequestration and water run-off and infiltration [58]. Some researchers have observed lower adaptive capacity in drier zones exhibited in the form of population’s more limited coping strategies, which in turn may contribute to increased VBD risk [59].

### Changing livelihoods/land use and increased vulnerability

Historically, as already suggested above, pastoralists in arid and semi-arid drylands relied on mobility and migration as an adaptive strategy to cope with low and highly variable rangeland productivity [12, 49, 60]. However, access to productive rangeland has shrunk, while pastoralist, and thus livestock populations, have grown along with land use conflicts. Exclusion from, or limitations placed on traditional livelihood and cultural practices have accompanied the establishment and increased enforcement of boundaries of protected areas, cropland expansion made possible through irrigation infrastructure development, and privatization of formally communal lands. These factors, and the increased land use conflicts have pushed pastoralists into dryer, more marginally productive rangelands. These marginal areas often include habitat for arthropod vectors (e.g. tsetse flies) and wildlife serving as reservoirs for zoonotic disease (e.g., trypanosomosis) [61].

The presence of livestock herds on the edges of, or encroaching into, protected areas increases the risk of pathogen spill-over from wildlife. This is illustrated by the case of (non-vectorial) transmission of bovine tuberculosis in Sub-Saharan Africa [62], and could also be the case for several vector-borne parasites from wildlife. This has been little studied despite their zoonotic potential, such as that of filarids from wild mammals (e.g., *Onchocerca* spp., *Dipetalonema* spp. and *Loaina* spp.) [26].

Other factors indirectly associated with changing livelihood and land use may contribute to dryland population’s increased vulnerability to VBDs and climate change. Social inequality and political marginalization of certain dryland groups has been shown to increase disease risk [63]. Similarly, poorer health among dryland people in general is associated with a lack of political voice or ability to negotiate power relations, and limited access to resources, technologies and networks [49]. For instance, increased exposure of pastoralists, hired herders and inhabitants of poor villages to Rift Valley fever vectors is often unaccounted for when irrigation schemes are sited nearby, while others pushed into marginal lands are at increased VBD risk as described above.

Sedentism, urbanization, and the livelihood shifts they imply in sub-Saharan Africa, as well as in other less-developed parts of the world, are also strongly associated with increasing incidence of obesity, diabetes, cardiovascular disease and other non-communicable diseases [64]. This, in turn, can increase communicable disease risk, including that of VBDs. For example, the link between malaria and non-communicable diseases has been documented in several reports including a case-control study of 1466 urban adults in Ghana. This study found that patients with type 2 diabetes had a 46% increased risk for infection with *Plasmodium falciparum*. Thus, increasing diabetes may contribute to malaria risk [65]. This health transition involving the double burden of communicable diseases, including VBDs, and noncommunicable diseases apparently related to livelihood shifts and urbanization, is increasingly being seen in Africa [66, 67].

Yet the effects of modernization on vulnerability can be unevenly distributed within the same pastoralist societies. Studies from Africa and Mongolia found that wealthy herders have access to better pastures, while poorer families are being pushed into increasingly marginal areas [49]. In semi-arid Central Asia, East Africa and the Sahel, sedentarization policies and changes in rangeland management, while providing better access to education and health infrastructures for some pasturalists [49], have increased social vulnerability for others [60].

It follows that improving the control and prevention of VBDs requires a better understanding of the changes taking place in the structure and dynamics of dryland societies. Given their formerly high degree of internal cohesion, self-organization, and traditional ecological knowledge [12], it is likely that dryland communities' indigenous health systems incorporated disease surveillance. Thus, VBD interventions incorporating indigenous environmental indicators offer a novel, social-ecological systems approach to community-based VBD outbreak risk forecasting [68]. Traditional knowledge among dryland cultures also has been shown to enable pastoralists to control, manage and treat parasitic and other illnesses. The Maasai, for instance, use a diversity of herbs and plant parts, in various remedies to treat common ailments such as malaria, skin disease, diabetes, cough and parasitic infections with self-reported effectiveness ranging from 52% in the case of skin diseases to 60% and up to 70% in the case of malaria and diabetes respectively [69].

### Drylands as complex social-ecological systems

Understanding the strategies used for dealing with dryland environmental constraints and uncertainty, particularly in relation to biodiversity and climate variability, has advanced significantly. This includes understanding the dynamics of ‘coupled human-natural systems’ taking into account human and natural ecologies, and the multiple layers of interaction among them (e.g. [20, 70]) applied specifically to drylands systems [71, 72]. In fact, discovery of an intricate, complex, and dynamic relationship between resource management systems and dryland ecosystems contributed importantly to the development of complex social-ecological systems as a body of theory and practice [73].

In addition to studies focused specifically on dryland societies already mentioned above, directly applicable to VBDs and their control, we found notable syntheses describing drylands as exemplifying the utility of SESR framing for elucidating how linked human-natural systems work; that is, retain their functionality or become dysfunctional in terms of meeting human needs and desires [11, 74, 75].

SESR represents a formal elaboration of the idea of human societies as embedded in and as part of ecosystems, forming a “whole” consisting of human social and natural ecological subsystems [76]. The integrated human and natural systems that constitute a so-called coupled human-natural system themselves consist of many interacting components, as subsystems of subsystems that make up the “whole.” This perspective and associated body of theory developed in response to environmental and resource problems being perceived as ‘complex systems problems’ which call for more creative forms of collaboration between scientists and society at large (particularly stakeholder groups, or those most affected), involving a broader range of disciplines, skills and participation [77].

**Fig. 2:**
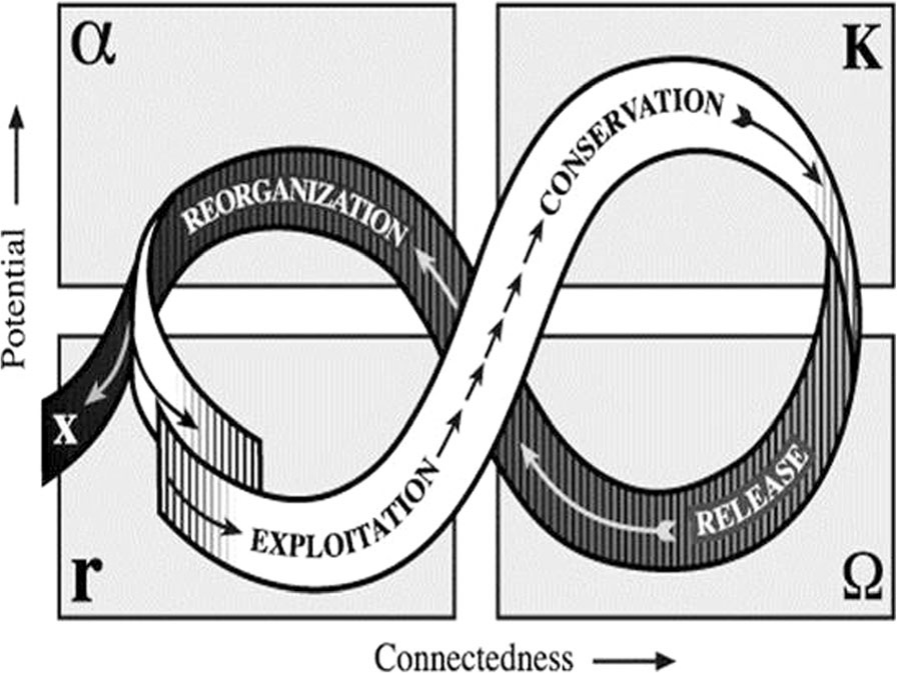
The adaptive cycle showing how changes in social-ecological systems characteristically exhibit two phases. Following collapse, a system can repeat the cycle (the white path) or transform into another system of different structure and function (the black path). A resilient system—i.e., one less vulnerable to unexpected shifts or collapses with undesirable or even catastrophic consequence for human populations—may successfully navigate itself through each of the phases and into new regime that satisfies societal goals. That is, it need not return via the α → r transition as before a crisis, thus repeat the cycle. In general, however, successful navigation (an indication of resilience) suggests the capacity to recognize barriers, critical thresholds and principles associated with this front loop that can trap a system—resulting in a pathology. System features allowing escape from these traps have been provisionally described [78]—representing adaptive management

At the core of SESR as a theory of change is the “adaptive cycle”, described as metaphorical, analytical tool for understanding social-ecological systems. Numerous studies of a wide range of “managed” and unmanaged or primarily natural ecosystems tend to follow the “figure 8” pathway characterized by two phases: exploitation (growth) and conservation, and release (or collapse) and reorganization as shown in Fig. 2. The adaptive cycle explains why historically, in most cases, management efforts have failed (e.g., most managed fisheries have either collapsed or have been over-fished, and efforts to control floods or pests frequently have resulted in worse floods or pest outbreaks). Common to all these is the aim of controlling a target variable (e.g., an insect pest) typically top down and with limited consideration of underlying changes taking place in the system over time [21].

Adaptive cycles are cycles exhibited by human systems and natural systems, as coupled human-natural systems, at multiple levels of organization: individuals, communities, watersheds or river basins, and ultimately, globally speaking in theory, the biosphere. These cycles are exhibited by each of the smaller scale entities (subsystems) nested in the larger ones. The complexity of living systems is largely a manifestation of this nested, hierarchical structure and associated dynamics involving interactions between levels (representing different space and time scales). The adaptive cycle has proven useful for revealing how larger scale dynamics (e.g., land use and climate change) interact with smaller scale dynamics (e.g., revolts or insect outbreaks) to produce unexpected consequences. It can also be used for retrospective analysis to investigate  why and how abrupt and even catastrophic changes occur [20, 38, 78].

SESR has been found particularly applicable to rural settings where traditional knowledge and perspectives is integral to adaptive capacity [79]. Not surprisingly resilience studies in drylands have reported a strong social-ecological coupling [12, 75, 80] along with other features characteristic of small-scale societies. SESR framing is particularly well-suited to analysis the complex interplay between dryland environments, vectors of zoonotic parasites (e.g., ticks, fleas, black flies, mosquitoes and sand flies) and their relationships with humans, which may result in the spread of bacteria, viruses, protozoa or helminths [26].

### Local traditional knowledge and biodiversity

Dryland societies’ nomadic, semi-nomadic, transhumant and sedentary smallholder agricultural livelihoods all entail a deep knowledge and understanding of the environment and its management [12, 16, 23–25]. This includes knowledge of the periodicity (seasonality, wildlife migrations, etc.) as well as unpredictability of natural events, and extends to the variety and variability expressed by the biodiversity unique to drylands.

Despite the aridity, a remarkable variety of genotypes, species, and communities of plants and animals have adapted, naturally or through selective breeding by dryland farmers and pastoralists, to the scarcity of water and extreme and unpredictable environmental conditions. These wild and “natural”, semi-domesticated and domesticated plants and animals represent an inestimably valuable source of adaptive evolutionary potential. Traditional dryland society’s resourcefulness and resilience, including its role in mitigating disease transmission in some cases [81, 82], is largely a consequence of this biodiversity that unfortunately is rapidly eroding [83].

**Fig. 3:**
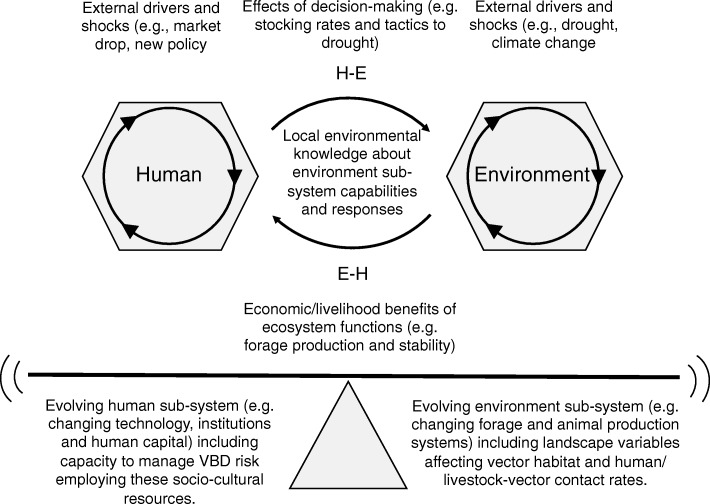
Adaptive management of Vector-borne disease (VBD) risk in a pastoral grazing system. The figure shows the linkages between social and ecological aspects as uncovered by dryland researchers, with VBD transmission added by the work reported in the present study. The economic/livelihood benefits of ecosystem functions can be extended to include the mitigation of VBD transmission associated with landscape function. Similarly, the local knowledge of Human-Environment (H-E) interactions include how livestock management decisions in consideration of external drivers effect landscape function associated with VBD transmission. (Modified from [75], Fig. 8.7)

Understanding how these societies exploit and manipulate biodiversity sustainably is key to understanding the resilience of dryland social-ecological systems particularly in light of increasing threats associated with climate change [84]. Stafford-Smith et al. elaborated on how dryland traditional and modern grazing systems function to maintain resilience [75]. This could best be described as a coevolutionary “dance” in which pastoralists keep step with a continually changing environment, accounting for risks while seeking to maximize benefits in the form of livestock production (Fig. 3). Behind much of the dynamic behavior of this dryland system is the idea of ‘landscape function’, which reflects and produces the variation expressed in the variety of species of plants, animals, and microbes. Managing VBD outbreak risk can be included in this scheme, in the context of ‘landscape function,’ as noted in Fig. 3.

Landscape function is described as the capacity of a landscape to regulate nutrients and water and toconcentrate them in vegetated patches where soil biota maintain nutrient cycles and water infiltration, impeding runoff thus soil erosion [75]. Vector species are of course a component of this system, and their changing distributions and abundances regulated by it. Loss of landscape function is the loss of this variability and increased homogeneity, thus dysregulation potentially including that of vector abundance.

### Social-ecological system resilience and vector-borne disease transmission

Resilience as a dimension of stability of complex systems and its application to vector borne diseases was first suggested by Holling [85] and later by Holling and colleagues [21]. This was expressed as an example of the adaptive cycle to help explain a top-down, command and control approach to vector control as a social-ecological system “pathology”.

Holling and colleagues cited malaria resurgence as an example from their perspective as ecologists [21]. They point out how insecticides and anti-malarial drugs used to control transmission typically achieve success, but only initially. The success reinforces the commitment to this top down approach (e.g. limited community involvement in vector control), while chemical and drug resistance eventually appears in the vector and parasite populations. Meanwhile, the proportion of susceptible humans has grown as a result of reduced transmission. As a consequence, the risk of an outbreak has increased while the ability to control it decreases.

This progression corresponds to the first two phases of the adaptive cycle and a “pathology of disease control” [29] in which institutions become increasingly inflexible (conservative) after initial success in controlling a disease, followed by a period of denial as warning signs go unheeded until a crisis develops. A remarkable feature of the adaptive cycle is its demonstration of how disease emergence results from the interaction of variables on vastly different time and space scales [29, 36, 86]. In the malaria case, this refers to the small and fast dynamics involving mosquito and parasite population ecology and genetics. This contrasts with the large and slow dynamics involving susceptibility (change in herd immunity in the human population). In this example, the disease system exhibits resilience.

As described above, the release and reorganization phase of the adaptive cycle represents how a social-ecological system may (depending largely on the human institutional response) undergo a transformation to a more desirable system regime. In the case of malaria this would be one of sustainable control or elimination. The extensive body of SESR-framed work offers the potential for far more elaboration of the implications for adaptive VBD control.

These ideas remain to be imported into VBD research and interventions, as it requires bridging the relatively large disciplinary gap separating biomedical science and ecology, as also found for biomedicine and social sciences [87]. Waltner-Toews was the first in the biomedical research community to suggest emerging infectious diseases as representing failures “to understand the socio-ecological systems we live in, and failures to respond to new understandings as they are uncovered” [88]. Subsequently, others specifically described the applicability of SESR framing and how zoonotic and VDB transmission dynamics, particularly the current era of emerging and re-emerging infectious diseases, is largely driven by land use change (i.e., urbanization, agriculture intensification, deforestation) affecting host-parasite dynamics at the landscape level [30, 31, 89].

In addition to these studies pointing to VBD emergence as proximally a consequence of landscape level dynamics several others have specifically drawn on principles developed in landscape ecology. This ecology subdiscipline is particularly relevant to understanding VDB transmission dynamics thus control. Continually changing land use mosaics viewed at the scale of human interaction (typically hectares or multiple square kilometers in the case of pastoralist movements), including varying physical structure and processes involving abiotic and biotic components, is the central focus of the field of landscape ecology [90]. A number of researchers have explored the role of landscape change in relation to VBD transmission or risk [91–93], including most notably Pavlovsky [94] who coined the term landscape epidemiology much earlier. Development of this idea thus did not have the benefit of SESR. Though Bradley’s [91] description of ‘chronotones’ closely aligns with the cyclic, dynamic nature of changing vector habitats.

In many ways the issue of VBDs and climate change in drylands epitomizes the challenges and opportunities suggested by the SESR frame globally. Dryland systems viewed from the SESR perspective offer the potential for exemplary models of adaptive management that combine VBD control and climate change adaptation. It remains a matter of their incorporation within programmes based on principles already articulated for sustainable dryland agricultural, pastoral, and mixed agro-pastoral systems [e.g., [74, 95].

### The way forward: operationalizing SESR for VBD interventions

SESR-framed drylands research has revealed how dryland ecosystems, either with minimal human activity or those exploited by traditional pastoralist societies, maintain relatively high levels of resilience. This contrasts with numerous cases in which social-ecological system dysfunction (i.e., a systemic pathology exhibited as repeated episodes of degradation) where resilience has been compromised. This includes the adaptive governance of VBDs, climate change adaptive capacity as well as that required to manage the potential for climate change to exacerbate VBD threats.

A re-envisioned “drylands development paradigm” largely based on SESR and dryland’s climatic unpredictability among dryland’s other distinct features—resource scarcity, remoteness from markets, and distance from centers of governance—has been articulated by Reynolds, Stafford Smith and colleagues [11, 75, 96]. It logically follows these principles are equally applicable to VBD control, particularly taking into account linked climate-VBD dynamics, and thus should be in such intervention efforts.

These principles are:

- *The coevolutionary nature of social and ecological systems, such that system collapse principally occurs when this relationship becomes dysfunctional, not just because of change.*
- *The need to focus very carefully on the appropriate slow variables and their thresholds in order to determine the state of this coevolutionary system as a matter of particular importance in variable environments.*
- *The massive effect that cross-scale interactions can have on dryland systems that are usually particularly poorly equipped to deal with these because of their distant voice.*
- *The vital importance of the right shared mental models in the form of local knowledge at a variety of scales for maintaining the functionality of the coupled system—particularly important in drylands where variability slows down experiential learning.*

In the context of dryland pastoralist systems specifically, whether of traditional pastoralists or others dependent on rangeland livestock liveilhoods, landscape function appears most critical to understanding the host-vector-environment epidemiological triad. Thus, the schema illustrated in Fig. 3 conceptualizes a hypothetical adaptive VBD management programme, including consideration of linked climate-VBD dynamics, for a dryland pastoral system.

The translation of this, and similar SESR-based schemata for other livelihood modes and of the above principles into operational criteria is a crucial next step. This includes explicit protocols tailored to needs of VBD intervention planners and managers. The absence until recently of such criteria even if generic but including explicit guidelines and/or protocols (i.e., generally applicable to all bioclimatic zones, biome types, or ecosystems) has been a major factor limiting application of the SESR frame including the ‘ecosystem approach to health’ [34]. A separate, small but growing literature has developed suggesting the application of SESR to climate change adaptation (e.g., [97–99].

Other methodological advances are needed that are aimed at facilitation of cooperative knowledge generation toward strengthening adaptive capacity locally, building on recent efforts conducted outside drylands [100, 101]. Inclusion of consideration of cross-scales influences, considered a critical determinant of resilience in social-ecological systems should be added. For drylands specifically a promising example is that aimed at enabling multi-level participation, as a basis for strengthening adaptive capacity through cooperative knowledge generation, as carried out with Gabra pastoralist communities of Northern Kenya [102].

Perhaps most challenging and a critical missing piece is collaborative, iterative design and refinement of indicators that can be used to monitor and evaluate performance of SESR framed VBD interventions. SESR-based monitoring and evaluation (M&E) methods and tools are needed. These must be capable of evaluating not only inputs-outputs and outcomes, but also processes (e.g. empowerment), behavioral change patterns and economic dimensions during and post VBD intervention. Several elaborate M&E frameworks have been or are being developed for climate change adaptation and resilience [103]. Current efforts are underway to apply these to VBD interventions in Africa drylands and Southeast Asia (Ramirez, unpublished).

## Conclusions

This review found an absence of published literature describing the application of SESR to VBD control, or VBD control combined with climate change adaptation in drylands. Yet it found a significant drylands literature including studies and applications of SESR addressing topics, issues, and common themes bearing directly on vector-borne disease control and climate change adaptation consistent with the SESR perspective.

Remarkable advances should be possible based on what could be characterized as scientific break-throughs in both understanding drylands as complex social-ecological systems and in the development of frameworks for research and intervention aligned with the social-ecological systems theory. There is a small but growing literature on climate change adaptation including studies describing intervention approaches employing SESR, particularly its conception of resilience.

A literature does not yet exist describing intervention-oriented research that involves community-based participatory research and practice that combines climate change adaptation and vector-borne disease control. Evidence from studies across separate literatures strongly suggests a significant untapped potential in this regard. Thus, further development and testing of transdisciplinary-participatory action research methods around knowledge, both formal and local or traditional, of meteorology, human and vector ecology, and landscape dynamics should be a priority.

The over-emphasis on disciplinary research and disincentives for working across disciplinary boundaries, which historically have impeded interdisciplinary research, obviously represents a substantial challenge. Yet, as evidenced by the literature reviewed here, the study of drylands as complex social-ecological systems offers an integrative agenda that is beginning to trigger such a transdisciplinary research programme.

## Additional file

Additional file 1: Multilingual abstracts in the five official working languages of the United Nations. (PDF 545 kb)

## Abbreviations

M&E: Monitoring and evaluation
SESR: Social-ecological systems and/or resilience theory
VBD: Vector-borne disease

## Authors’ information

Bruce A. Wilcox received his undergraduate and graduate education from University of California at San Diego (BA, biology; PhD population biology) and Yale University (MS, ecology and evolutionary biology). A founder of the field of conservation biology and the journal *Conservation Biology*, he also was the founding editor of EcoHealth and first Chief Executive of the International Association for Ecology and Health. His current research focuses on integrative research and its applications in environmental health, tropical disease research, and global health. He is a frequent consultant and advisor to international agencies including the World Health Organization on ecological and complex systems applications to environmental health challenges, particularly tropical disease prevention and control.

Michel de Garine-Wichatitsky has a backgroung in Veterinary Medicine (DVM, Ecole Nationale Vétérinaire de Maisons-Alfort) and received his post-graduate education in Ecology at the University of Montpellier in France (MSc, PhD, HDR). He is a Senior Researcher for the French agricultural research and international cooperation organization CIRAD, currently based in Bangkok with the Faculty of Veterinary Medicine of Kasetsart University, and Research Associate with the Department of Biological Sciences, University of Zimbabwe. His career has been devoted to research, higher education and management of biodiversity and health in tropical regions, including South-East Asia, Southern Africa and the South Pacific. Michel has embraced multiple aspects of “wildlife-livestock-human interfaces” and enthusiastically adopted multi- and inter-disciplinary approaches associating ecology, veterinary medicine, anthropology, parasitology and epidemiology.

Bernadette Ramirez, Ph. D, Provides support for and coordinates projects studying population health vulnerabilities to vector-borne diseases and ways to increase population resilience against the impact of climate change in Africa and southeast Asia. Bernadette joined WHO-TDR in June 2007 with over 20 years of academic and research experience in health product innovation, drug discovery, vaccines and the development of diagnostics for infectious tropical diseases.

Pierre Echaubard, Ph. D, is a health ecology researcher. He specializes in infectious diseases ecology and evolution, vector-borne diseases adaptive management and the linkages between biodiversity and health. He is currently focusing on operationalizing protocols applying Social-Ecological Systems approaches to VBD control and health development in Myanmar, Cambodia, Thailand and Lao PDR, including developping indicators assessing outcomes and processes in relation to community engagement, adaptive capacity building and resilience. He has academic experiences and postgraduate degrees from the University of Montpellier, France, the ETH Zurich, Switzerland and a PhD in infectious disease ecology and evolution from Laurentian University, Canada. He is associate editor of the journals Infection Genetics and Evolution (Elsevier) and guest editor for Evolutionary Applications (Wiley).
