# Supplementary material for: Vector-borne disease and climate change adaptation in African dryland social-ecological systems
Source: Infect Dis Poverty. 2019 May 27;8:36. doi: 10.1186/s40249-019-0539-3 (PMC6535848; doi:10.1186/s40249-019-0539-3)
Supplement: Supplementary file 1 — Multilingual abstracts in the five official working languages of the United Nations. (PDF 545 kb) [file 40249_2019_539_MOESM1_ESM.pdf]

Translation of the abstract into the five official working languages of the United Nations

الأمراض المحمولة بالنواقل والتكيف مع تغير المناخ في الأنظمة البيئية-الأجتماعية للأراضي الجافة في إفريقيا

Wichatitsky, Bernadette Ramirez-Bruce A. Wilcox, Pierre Echaubard, Michel de Garine

نُبذة

لمحة عامة: تشكل الأراضي الجافة ، التي تعد من بين النظم البيئية الأكثر تغيراً طبيعياً وبيئياً في المحيط الحيوي ، ثلاثة أرباع القارة الأفريقية. ونتيجة لذلك ، كانت الاستدامة البيئية والتنمية البشرية جنباً إلى جنب مع مكافحة الأمراض المحمولة بالنواقل (BDV) تاريخياً تحدياً على وجه الخصوص في أفريقيا ، لا سيما في الأراضي الجافة الواقعة جنوب الصحراء والساحل. هنا ، عبء الأمراض المحمولة بالنواقل ، وانعدام الأمن الغذائي ، وتدهور البيئة ، والضعف الاجتماعي شديدة بشكل خاص. يمكن أن يؤدي تغير المناخ إلى تفاقم التهديدات الصحية البيئية في أفريقيا ، التي أصبحت أبعادها الاجتماعية الآن جزءاً من جدول أعمال التنمية الدولية. وبناءً على ذلك ، فإن الحاجة إلى فهم التحركات والاقتران المعقّد بين المجموعات السكانية والبيئات على نحو أفضل كما يتضح في الأراضي الجافة يُعترف به بشكل متزايد على أنه ضروري لتصميم تدخلات أكثر استدامة.

الفكرة الرئيسية: يدرس هذا الاستعراض الاستطلاعي تحدي مكافحة الأمراض المحمولة بالنواقل في الأراضي الجافة مع التركيز على أفريقيا ، والتغيرات المناخية والبيئية والاجتماعية المستمرة الجارية. استمرت مجتمعات الأراضي الجافة وازدهرت في الماضي على الرغم من تغير المناخ ، والطقس المتطرف وغير المتوقع ، والظروف الهامشية للزراعة. ومع ذلك ، فقد ساهمت القوى المتداخلة إلى حد كبير خارج عن سيطرة المجتمعات التقليدية في الأراضي الجافة ، إلى جانب الآثار السلبية للعولمة ، في تآكل الموارد الثقافية والطبيعية في الأراضي الجافة. وقد أدى ذلك إلى فقدان المرونة الكامنة وراء القدرة التكيفية التي كانت معروضة في السابق على نطاق واسع بين مجتمعات الأراضي الجافة. وتوضح مجموعة متزايدة من الأدلة المستمدة من الدراسات الخاصة بإدارة الموارد الطبيعية والبيئية كيف أن هذه العوامل والتدخلات بشكل تنازلي يمكن أن تعوق ، في ضوء التعقيد المتأصل في نظام الأراضي الجافة ، التنمية المستدامة والتحكم في الأمراض المحمولة بالنواقل. إن تعزيز القدرة على التكيف بما في ذلك من خلال الطرق المجتمعية والقائمة على المشاركة والتي تعتمد على المعرفة المحلية ومصممة خصيصًا للظروف البيئية المحلية ، هي أفضل ما يمكن أن يكون لعكس الاتجاهات الحالية.

الأستنتاج: توجد فرصة كبيرة للتصدي على التهديد المتزايد للأمراض المحمولة بالنواقل وتغير المناخ من خلال طرق تهدف إلى تعزيز القدرة على التكيف. يوفر الإطار والأساليب التكاملية القائمة على النظم البيئية الاجتماعية ونظرية المرونة مجموعة جديدة من الأدوات التي تسمح بمعالجة التهديدات المتعددة ومصادر الضعف معا. ويمكن أن يساعد تكامل التقدم الأخير في علم بيئة الأمراض التي تحملها ناقلات الأمراض ونشر هذه الأدوات على نطاق أوسع في عكس الاتجاهات الاجتماعية والبيئية السلبية السائدة حالياً في الأراضي الجافة في أفريقيا.

Translated from English version into Arabic by Mohamed Shawkat, proofread by Aalya Al-Beeshi, through

TRANSLATORS
WITHOUT BORDERS

非洲干旱地区社会生态系统中媒传疾病及其对气候变化的适应

Bruce A. Wilcox, Pierre Echaubard, Michel de Garine-Wichatitsky, Bernadette Ramirez

摘要

引言：干旱地区是生物圈最受自然条件限制和环境变化最多的生态系统之一，占非洲大陆的四分之三面积。因此，在非洲，尤其是撒哈拉以南和萨赫勒干旱地区，环境可持续性，以及与媒传疾病（VBD）防治相关的人类发展极具历史挑战性。其中，VBD 负担、粮食安全、环境退化和社会脆弱性尤为严重。气候变化可能导致非洲环境健康造成的威胁加剧，其社会层面的问题现已成为国际发展议程的一部分。因此，人们越来越认识到需要更好地了解人口和环境（如干旱地区）的动态变化，以及复杂的相互作用，这对于设计更可持续的干预措施至关重要。

正文：本文综述了非洲干旱地区防治媒传疾病面临的挑战，以及正在发生的、巨大的环境和社会变化。在过去的一段时间里，尽管出现了气候变化，如极端和不可预测的天气以及农业的边际条件的不断变化，干旱地区的社会仍然繁荣昌盛。然而，不受传统干旱地区社会控制的侵扰力以及全球化的负面影响导致了干旱地区文化和自然资源受到侵蚀。这导致以前在干旱地区社会中广泛展现出的适应能力丧失其弹性。来自环境和自然资源管理研究得到的诸多证据表明，鉴于干旱地区系统固有的复杂性，这些因素和自上而下的干预措施将阻碍可持续发展和媒传疾病的防治工作。通过基于社区的参与式方法来提高适应能力，尤其是要基于当地文化，并根据当地生态条件进行调整，最有希望扭转当前的趋势。

结论：采用旨在提高适应能力的方法，可以同时应对媒传疾病和气候变化引起的日益严重的威胁。建立基于社会生态系统和弹性理论的综合框架和方法，可以综合性地解决由多种威胁和脆弱性带来的问题。将媒传疾病生态学的最新进展与上述方法相结合，有助于扭转目前出现在非洲干旱地区的社会和环境的负面趋势。

Translated from English version into Chinese by Cong-Shan Liu, edited by Pin Yang

CHINESE CENTER FOR DISEASE CONTROL AND PREVENTION
NATIONAL INSTITUTE OF PARASITIC DISEASES

Maladies vectorielles et adaptation au changement climatique dans les systèmes socio-écologiques des zones arides africaines

Bruce A. Wilcox, Pierre Echaubard, Michel de Garine-Wichatitsky, Bernadette Ramirez

Résumé

Contexte: les zones arides, constituant les trois quarts du continent africain, comptent parmi les écosystèmes de la biosphère les plus limitants naturellement et, d’un point de vue environnemental, les plus variables. En conséquence, la durabilité de l’environnement et le développement humain ainsi que la lutte contre les maladies vectorielles ont toujours constitué un défi particulier en Afrique, notamment dans les zones arides subsahariennes et sahéliennes. Ici, la charge de morbidité due aux maladies vectorielles, l’insécurité alimentaire, la dégradation de l’environnement et la vulnérabilité sociale sont particulièrement graves. Le changement climatique peut exacerber la légion de menaces pour la santé liées à l’environnement en Afrique, dont les dimensions sociales font désormais partie du programme de développement international. En conséquence, la nécessité de mieux comprendre la dynamique et le couplage complexe des populations et des environnements, illustrés par les zones arides, est de plus en plus reconnue comme essentielle à la conception d’interventions plus durables.

Corps principal: cet examen exploratoire passe en revue le défi que représente la lutte contre les maladies vectorielles dans les zones arides en mettant l’accent sur l’Afrique, ainsi que les changements environnementaux et sociaux dramatiques en cours. Les sociétés des zones arides ont persisté et même prospéré dans le passé, malgré les changements de climat, les conditions météorologiques extrêmes et imprévisibles et les conditions marginales pour l'agriculture. Cependant, des forces intrusives échappant largement au contrôle des sociétés des zones arides traditionnelles, ainsi que les impacts négatifs de la mondialisation, ont contribué à l’érosion des ressources culturelles et naturelles des zones arides. Cela a conduit à la

perte de résilience sous-jacente à la capacité d'adaptation, autrefois largement répandue parmi les sociétés des zones arides. De plus en plus de preuves provenant d’études sur la gestion de l’environnement et des ressources naturelles montrent comment, compte tenu de la complexité inhérente aux systèmes de zones arides, ces facteurs et ces interventions descendantes peuvent entraver le développement durable et la lutte contre les maladies vectorielles. Le renforcement de la capacité d’adaptation, notamment par le biais de méthodes participatives basées sur la communauté qui s’appuient sur les connaissances locales et sont adaptées aux conditions écologiques locales, constitue le meilleur moyen d’inverser les tendances actuelles.

Conclusions: il existe une opportunité significative de traiter simultanément la menace croissante des maladies vectorielles et du changement climatique grâce à des méthodes visant à renforcer la capacité d’adaptation. Le cadre et les méthodes d’intégration basés sur les systèmes socio-écologiques et la théorie de la résilience offrent un nouvel ensemble d’outils permettant de traiter simultanément de multiples menaces et sources de vulnérabilité. L’intégration des avancées récentes en matière d’écologie des maladies vectorielles et le déploiement plus large de ces outils pourraient contribuer à inverser les tendances sociales et environnementales négatives actuellement observées dans les zones arides africaines.

Translated from English version into French by Iris Soliman, proofread by Ingrid Vieira Mary, through

TRANSLATORS
WITHOUT BORDERS

Трансмиссивные болезни и адаптация к изменению климата в социально-экологических системах засушливых районов Африки

Брюс Э. Уилкокс, Пьер Эшубар, Мишель де Гарин-Уичатитски, Бернадетт Рамирез

Аннотация

Общие сведения: Засушливые районы, представляющие собой экосистемы с неблагоприятными и непостоянными климатическими условиями, занимают три четверти Африканского континента. Поэтому обеспечение экологической устойчивости и развитие человеческого потенциала, а также борьба с трансмиссивными болезнями в Африке, особенно в засушливых районах к югу от Сахары и в Сахеле, всегда представляли особую сложность. Здесь наиболее высоко бремя трансмиссивных болезней и наиболее остро стоят такие проблемы, как отсутствие продовольственной безопасности, деградация окружающей среды и социальная уязвимость. Изменение климата может усугубить многочисленные угрозы здоровью населения Африки, социальные аспекты которых теперь входят в международную повестку дня в области развития. В этой связи всё более распространенным становится мнение, что для разработки последовательных и рассчитанных на долгосрочную перспективу мер, необходимо детальнее изучить динамику взаимодействия и сложную систему связей между населением и окружающей средой на примере засушливых районов.

Основная часть: Настоящий аналитический обзор посвящен проблеме борьбы с трансмиссивными болезнями в засушливых районах, в первую очередь в Африке, а также происходящим там серьезным экологическим и социальным переменам. В прошлом население засушливых районов сохранялось и даже процветало, несмотря на изменения климата, экстремальную и непредсказуемую погоду и неблагоприятные условия для сельского хозяйства. Однако внешние факторы, не поддающиеся контролю со стороны местного населения засушливых

районов, а также негативные последствия глобализации способствовали истощению культурных и природных ресурсов в этих регионах. Это привело к снижению сопротивляемости, лежащей в основе адаптационного потенциала, который ранее был свойственен сообществам засушливых районов. В ходе исследований процессов управления окружающей средой и природными ресурсами появляется всё больше сведений, свидетельствующих о том, что с учетом сложного характера экосистемы засушливых районов эти факторы, а также вмешательство сверху, могут препятствовать устойчивому развитию и борьбе с трансмиссивными болезнями. Укрепление адаптационного потенциала, в том числе с помощью методов, обеспечивающих участие местных жителей и опирающихся на их опыт, а также учитывающих местные экологические условия, является наиболее перспективным способом преодоления нынешних тенденций.

Выводы: Существует реальная возможность противодействовать растущей угрозе трансмиссивных болезней и одновременно последствиям изменения климата с помощью методов, направленных на укрепление адаптационного потенциала. Интегрирующие механизмы и методы, основанные на теории сопротивляемости социально-экологических систем, дают новый набор инструментов для комплексной борьбы с множественными угрозами и источниками уязвимости. Использование последних достижений в сфере экологии трансмиссивных болезней и широкое применение этих инструментов может способствовать преодолению негативных социальных и экологических тенденций, наблюдающихся в настоящее время в засушливых районах Африки.

Translated from English version into Russian by Daria Afanasyeva, proofread by Anna Krasnopeeva, through

Enfermedades transmitidas por vectores y adaptación al cambio climático en los sistemas socioecológicos de las zonas áridas africanas.

Bruce A. Wilcox, Pierre Echaubard, Michel de Garine-Wichatitsky, Bernadette Ramirez

Resumen

Introducción: las zonas áridas, uno de los ecosistemas de la biosfera más limitativos por naturaleza y variables según el medio ambiente, constituyen tres cuartas partes del continente africano. Como consecuencia, tanto la sostenibilidad medioambiental como el desarrollo humano, junto con el control de las enfermedades transmitidas por vectores (ETV), han sido un auténtico desafío a lo largo de la historia en África, sobre todo en las zonas áridas subsaharianas y sahelianas. Aquí, la carga de las ETV, la inseguridad alimentaria, el deterioro medioambiental y la vulnerabilidad social son especialmente graves. El cambio climático puede exacerbar las numerosas amenazas medioambientales a la salud en África, cuyo alcance social ahora forma parte del programa de desarrollo internacional. Por lo tanto, la necesidad de comprender mejor la dinámica y la compleja conexión de las poblaciones y los entornos como en el ejemplo de las zonas áridas se considera cada vez más crítica para diseñar actuaciones más sostenibles.

Texto principal: esta revisión de alcance examina el reto de controlar las enfermedades transmitidas por vectores en zonas áridas centrándose en África, así como los cambios sociales y medioambientales drásticos y en curso que tienen lugar. En el pasado, las sociedades de zonas áridas persistieron e incluso crecieron a pesar de los cambios climáticos, la meteorología extrema e impredecible y las condiciones marginales para la agricultura. Sin embargo, fuerzas intrusivas mayormente fuera

del control de las sociedades tradicionales de zonas áridas, junto con los efectos negativos de la globalización, han contribuido al desgaste de los recursos naturales y culturales de dichas zonas. Esto ha conducido a la pérdida de resiliencia subyacente a la capacidad de adaptación demostrada ampliamente con anterioridad entre las sociedades de zonas áridas. Un volumen creciente de pruebas obtenidas en estudios sobre la gestión de recursos medioambientales y naturales demuestra cómo, a la luz de la complejidad inherente de los sistemas de zonas áridas, estos factores e intervenciones descendentes pueden dificultar el desarrollo sostenible y el control de las enfermedades transmitidas por vectores. La mejor opción para revertir las tendencias actuales parece ser el fortalecimiento de la capacidad de adaptación, inclusive a través de métodos participativos comunitarios que se basen en el conocimiento local y se adapten a las condiciones ecológicas de la zona.

Conclusión: existe una oportunidad significativa para abordar simultáneamente la creciente amenaza de las enfermedades transmitidas por vectores y el cambio climático a través de métodos dirigidos al refuerzo de la capacidad de adaptación. El marco integrador y los métodos basados en los sistemas socioecológicos, así como la teoría de la resiliencia ofrecen un conjunto de herramientas novedoso que permite hacer frente a las múltiples amenazas y fuentes de vulnerabilidad en combinación. La integración de avances recientes en la ecología de las enfermedades transmitidas por vectores y un despliegue más amplio de estas herramientas podrá ayudar a revertir las tendencias sociales y medioambientales negativas que se observan actualmente en las zonas áridas africanas.

Translated from English version into Spanish by Carolina Forte, proofread by Mayra León, through

TRANSLATORS WITHOUT BORDERS
